# Systemic angiopoietin-1/2 dysregulation following cardiopulmonary bypass in adults

**DOI:** 10.4155/fsoa-2016-0072

**Published:** 2017-01-11

**Authors:** Emmanuel Charbonney, Elizabeth Wilcox, Yuexin Shan, Pablo Perez d'Empaire, Abhijit Duggal, Gordon D Rubenfeld, Conrad Liles, Claudia Dos Santos

**Affiliations:** 1 Centre de Recherche de l'Hôpital du Sacré-Coeur, University of Montreal, 5400 Boulevard Gouin Ouest, Montréal, QC H4J 1C5, Canada; 2 Interdepartmental Division of Critical Care, University of Toronto, University Health Network, 399 Bathurst St, Toronto, ON M5T 2S8, Canada; 3 Keenan Research Centre of Li Ka Shing Knowledge Institute, 30 Bond Street, Toronto, ON M5B 1W8, Canada; 4 Department of Anesthesia, University of Toronto 12th Floor, 123 Edward Street, Toronto, ON M5G 1E2, Canada; 5 Department of Pulmonary, Allergy, & Critical Care, Respiratory Institute, Cleveland Clinic Main Campus, 9500 Euclid Avenue, Cleveland, OH 44195, USA; 6 Interdepartmental Division of Critical Care, University of Toronto, Trauma, Emergency & Critical Care Program, Sunnybrook Health Sciences Centre, 2075 Bayview Avenue, Toronto, ON M4N 3M5, Canada; 7 Department of Medicine, University of Washington, 1959 NE Pacific St. Box 356420, Seattle, WA 98195, USA; 8 Interdepartmental Division of Critical Care, University of Toronto, St. Michael's Hospital-Keenan Research Centre of Li Ka Shing Knowledge Institute, 30 Bond Street, Toronto, ON M5B 1W8, Canada

**Keywords:** angiopoietin, biomarker, cardiac surgery, cardiopulmonary bypass, length of stay, vascular leakage, vascular permeability

## Abstract

**Aim:**

Vascular leakage following cardiopulmonary bypass contributes to morbidity. Angiopoietin-1 and -2 are biomarkers of endothelial dysfunction. Our aim was to characterize Ang-1 and -2 association with clinical characteristics and outcomes.

**Methods:**

Observational cohort study measuring Ang-1/-2 with a panel of cytokines in adults undergoing cardiopulmonary bypass.

**Results:**

Ang-2 levels increased immediately postop whereas Ang-1 levels decreased over time. No significant correlation was found with other inflammatory mediators. High correlation was found between the hospital length of stay and Ang-2 increase at 24 h (rho = 0.590; p < 0.0001). The predictors of Ang-2 increase were female gender, cross clamp time, transfusion of blood and absence of angiotensin-converting enzyme inhibitor as a pre-op medication.

**Conclusion:**

Angiopoietins can detect vascular leakage early and could impact patient's management to decrease length of stay after cardiac surgery.

## Lay abstract

Following cardiac surgery, patients develop swelling due to fluid leaking from their vessels into their tissues. We know that more swelling contributes to a longer stay in the hospital. The present study links a marker of vessel instability, namely angiopoietin (which we think could contribute to detect leakage), with patient's hospital length of stay. We also report patient's characteristics that are related to the release of angiopoietin. The measure of this marker after cardiac surgery could provide physicians with an alert signal and trigger treatments to minimize swelling that could impact on hospital stay.

**Figure 1.**
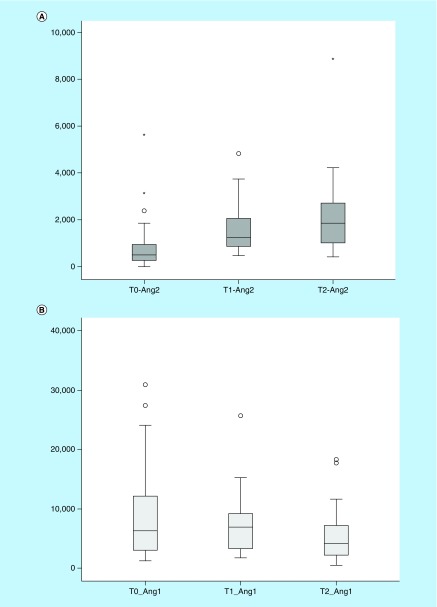
**(A)** Ang-2 increases significantly from T0 to T1 (p < 0.0001), but not from T1 to T2 (p = 0.063). **(B)** Ang-1 do not decrease from T0 to T1 (p = 0.196), but decreases significantly from T1 to T2 (p = 0.009). The symbol ° and * represent outliers.

**Figure 2.**
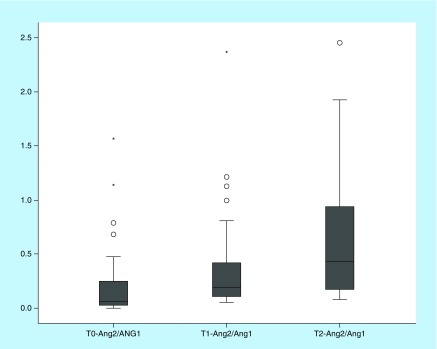
**The ratio increases from T0 to T1 (p < 0.0001), essentially due to early Ang-2 increase, and then it increases from T1 to T2 (p = 0.025) due to the drop of Ang-1.**

**Figure 3.**
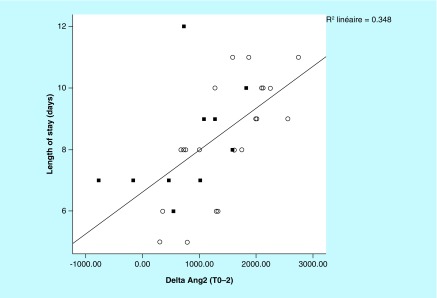
The patient hospital length of stay (LOS), including only those with LOS ≤14 days (n = 36), is highly correlated (rho =0.590; p < 0.0001) with the ang-2 increase (T0 to T2). Patients with pre-operative ACE inhibitors are displayed in black squares.

Systemic vascular leakage that occurs following cardiopulmonary bypass (CPB) is known to contribute to postoperative morbidity, including multiple organ failure, third space accumulation and increased length of stay (LOS) [1–4]. The pathophysiolocal process involves numerous pathways, including activation of coagulation, complement and complex inflammatory cascade [5–7]. Endothelium activation and dysfunction play important roles in leakage and point to the crucial role of endothelial activation in subsequent proinflammatory responses including activation of the coagulation cascade, extravasation of intravascular fluid into the extravascular space and enhanced leukocyte adhesion [8]. Despite the clinical relevance attributed to loss of intravascular compartmentation, methods to characterize and quantify endothelial dysregulation and vascular leakage in the clinical setting remains a significant challenge; any measure or marker able to overcome this challenge has the potential to impact clinical practice [9].

The importance of angiopoietin-1 (Ang-1) and -2 (Ang-2) in the regulation of endothelial quiescence and activation has recently been recognized and various studies have shown that; while Ang-1 functions to preserve vascular quiescence, Ang-2 mediates vascular activation and instability [10]. Recent studies have shown that the degree of endothelial dysregulation responsible for organ dysfunction might be associated to circulating levels of Ang-1, Ang-2 or their ratio [11]. These molecules are not only implicated in vascular biology but also in the regulation of endothelial permeability and may thus inform regarding the activation status of the endothelium [12]. In sepsis, for example, the balance of Ang-1 and Ang-2 is disrupted, and this disruption correlates with the degree of organ failure/injury and mortality [13]. Changes in circulating levels of Ang-1 and -2 are also associated with changes in other biomarkers (TNF, IL-6) of sepsis and endothelial activation [14,15].

The phenomenon of vascular inflammation and leakage that occurs in the context of cardiac surgery, with CPB, provides a unique experimental model in humans. Indeed, previous studies have reported increasing angiopoietin after cardiac surgery and its association with positive fluid balance, duration of mechanical ventilation, LOS in the intensive care unit (ICU) and respiratory failure [16–18]. Our aim was to characterize Ang-1 and Ang-2 changes and their association with preoperative and operative clinical characteristics, as well as with a panel of inflammatory markers.

## Methods

### Study population

The cohort has been described in detail previously and the present work is a secondary analysis of data with the addition of angiopoietin measurements [19]. We conducted a prospective observational study including nonconsecutive adult patients (age 18 years or older) undergoing elective, CPB surgery at an academic, medical center from September 2009 to 2011. Patients with active oral disease, infection, recent myocardial infarct (<2 weeks), pregnancy, immunodeficiency (including steroids or immunosuppressive agents <30 days) or autoimmunity, were excluded. The study received approval from the Institutional Review Ethic Board of Sunnybrook Health Sciences Centre (ON, Canada). Written informed consent was obtained preoperatively from all patients enrolled.

### Clinical data acquisition

Comprehensive clinical data was collected prospectively for the duration of the study, including: baseline demographics and characteristics, surgical procedure, CPB specifics, postoperative course and outcomes; the latter were mortality, delta creatinine, renal replacement therapy, ventilation duration and LOS (prolonged would be defined as >14 days) [20].

### Blood sampling

Baseline blood samples were obtained during preoperative blood test in the morning of surgery, or before surgery started (T0 = prior to CPB). Then sampling was done at admission to the ICU (T1) and 12–18 h later, postop day 1 (T2). Blood was collected in EDTA and citrate tubes. Samples were immediately centrifuged at 1700 × *g* at 4°C for 20 min, plasma was aliquoted in cryotubes and stored at -20°C, then later at -80°C until further analysis.

### Measurement of serum cytokines & mediators

Plasma concentrations (dilution factors indicated in parentheses) of Ang-1 (1:5) and Ang-2 (1:5) were measured by ELISA (R&D Systems Duoset kits, MN, USA) according to manufacturers’ instructions with optimizations: assays were performed in 50 μl per well; plasma samples were incubated overnight at 4°C and ELISAs were developed using Extravidin^®^-Alkaline Phosphatase (Sigma-Aldrich Canada Ltd, ON, Canada; 1:1000 dilution, 45-minincubation) followed by addition of p-nitrophenyl phosphate substrate (Sigma-Aldrich Canada Ltd) before optical density reading at 405 nm. Concentrations were interpolated from 4-parameter-fit standard curves. Background levels were determined from blank wells included on each plate (assay buffer added instead of sample), and the subsequent optical density was subtracted from all samples and standards prior to analysis. Samples with optical densities below the lowest detectable standard were assigned the value of that standard. Lower LODs for each assay were as follows: 19.53 and 27.34 pg/ml for Ang-1 and Ang-2, respectively.

In parallel, cytokine analyses were performed using Luminex mediator panel with Multiplexing immunoassay instrument (Luminex Technology, TX, USA). IL-1β, IL-2, IL-6, IL-10, IL-12, IL-17, IL-1 receptor antagonist, monocyte chemotactic protein-1, soluble intercellular adhesion molecule and granulocyte/macrophage colony-stimulating factor levels were determined. The sensitivities and details for the multiplex ELISAs were as per default parameters of the manufacturers. Each measurement (time point/individual) was performed in duplicate. Samples were analyzed randomly and laboratory personnel performing the analysis were blinded to group assignment.

### Statistics

Variables were described as means ± standard deviation when normally distributed or medians and interquartile range (IQR). Missing data points were excluded from the analysis. Given the type of explorative analysis, no power calculation was done. The Friedman Two Way Analysis of Variance was used for one-way repeated measures analysis of variance by ranks across multiple comparisons. Wilcoxon rank sum test was used to compare two samples between each other. Correlations were determined using Spearman rank correlation coefficient. Mann–Whitney was used for univariate analyses. Multiple regression model was used to determine predictive factors significant, with p-value <0.01 after univariate analysis. The sampling of patients was sufficient to explore a maximum of four variables in the model. Statistical analyses were performed using SPSS version 20 (SPSS, Inc, IL, USA). Differences were deemed significant if two-sided p-value was ≤0.05.

## Results

### Population course

We enrolled 41 patients in the study. Baseline and demographic characteristics are displayed in Table 1. The entire cohort was recruited at the same center and represents a homogenous population of elective CPB surgery. The type of anesthesia and extra-corporeal circuit were not recorded, while the type of surgery, the hospital course and outcomes are described in Table 2. The duration of mechanical ventilation was 1 day for the majority of patients (92%) and only one was ventilated longer than 3 days. Only three patients (7%) required vasopressor support over the first 24 h in the ICU and one patient had an intra-aortic balloon. All patients were discharged alive from the ICU and the hospital. Most (89%) patients were discharged directly home. For most patients, the hospital LOS was ≤14 days (n = 37; 90%).

### Temporal kinetics of circulating angiopoietin levels

Angiopoietin measurements were complete (3 time points) for 30 patients and partial (2 time points) for 11 patients; one sample was missing on T0, five on T1 and five on T2 (all different patients), indicating 91% (112/123) of available time points collected. Over the three time points studied, circulating Ang-2 levels (Figure 1A) increased (p < 0.0001; Friedman's test) while in parallel, Ang-1 levels (Figure 1B) decreased (p = 0.012; Friedman's test). The ratio of Ang-2/Ang-1 (Figure 2) also rose significantly over time (p < 0.0001; Friedman's test). The peak median values were on T1 for Ang-1 (6935 pg/ml, IQR 3192, 9263), on T2 for Ang-2 (1856 pg/ml; IQR 1004, 2737) and on T2 for Ang-2/Ang-1 ratio (0.43; IQR 0.16–0.97). When compared with baseline, Ang-2 levels and Ang-2/Ang-1 ratio were significantly higher at both post-CPB time (T1 vs T0 and T2 vs T0; all p < 0.0001). No difference was observed between T1 and T2 for Ang-2 (p = 0.063), but Ang-2/Ang-1 ratio still significantly increased (p = 0.025). Compared with pre-op levels (T0) Ang-1 was unchanged in the immediate postop period (T1; p = 0.196) but decreased significantly by the first postop day (T2 vs T0, p = 0.001; T2 vs T1; p = 0.009).

### Angiopoietins & other mediators

The increase in Ang-2 levels was accompanied by a significant increase in the levels of other proinflammatory mediators primarily in the immediate postop period (T1), including IL-1β (p = 0.004), IL-6 (p < 0.0001), monocyte chemotactic protein-1 (p < 0.0001), IL-10 (p < 0.0001), IL-12 (p < 0.0001) and IL-1 receptor antagonist; p < 0.0001), compared with pre-op (T0). Levels of granulocyte/macrophage colony-stimulating factor, soluble intercellular adhesion molecule, IL-2 and IL-17 were not found to differ from baseline. A trend for weak positive correlation was found between IL-6 at T1 and Ang-2 at T2 (rho =0.37; p = 0.38) and a nonsignificant inversed correlation between IL-6 at T1 and Ang-1 at T1 (rho =0.4; p = 0.17). No correlation was found between any other mediators and Ang level or changes over time. All values are shown in Table 3.

### Clinical associations

Regarding the effect of the surgical intervention and angiopoietin changes (delta), the cross-clamp time correlated with delta T0-T1 for Ang-2 (rho =0.41; p = 0.014, Spearman), as well as with delta T0-T2 for Ang-2 (rho = 0.44; p = 0.008), or with delta T0-T1 for the ratio Ang-2/Ang-1 (rho = 0.39; p = 0.021). The CPB time also correlated with the delta T0-T1 for the ratio Ang-2/Ang-1 (rho =0.35; p = 0.04).

For the postoperative course, an inverse correlation was found between delta creatinine T0-T1 and delta T0-T1 for the ratio Ang-2/Ang-1 (rho = -0.38; p = 0.027). No patient required renal replacement therapy. The mean hospital LOS was 8.4 ± 2.3 days. A significant correlation (rho =0.590; p < 0.0001) was found between hospital LOS (≤14 days) and delta Ang-2 for T0-T2, after excluding those with prolonged stay (Figure 3).

### Predictors of angiopoietin changes

No correlation was found with age, intraoperative temperature, or APACHE II and changes in the ratio of Ang-2/Ang-1 (delta T0-T1). Interestingly, being an active smoker (n = 5) significantly decreased observed changes (delta T0-T1) in the ratio Ang-2/Ang-1 as compared with nonactive smokers (n = 30), with medians of 0.033 (IQR **-**0.655, 0.068) and 0.129 (IQR 0.049, 0.407), smokers and nonsmokers, respectively (p = 0.027). No difference in baseline (T0) Ang-1, Ang-2 or ratio Ang2/Ang1 was found between active smoker versus nonactive smoker. A difference was also found for the delta ratio Ang-2/Ang-1 between male (n = 28) and female (n = 7) patients, with a median of 0.075 (IQ: 0.03, 0.24) as compared with 0.32 (IQ: 0.13, 0.87), respectively (p = 0.039). Patients reporting alcohol use (n = 5) were noted to have higher ratio Ang-2/Ang-1 at T2, than nonalcohol users (n = 31), with a median of 0.830 (IQ: 0.624, 1.491) as compared with 0.362 (IQ: 0.153, 0.880), respectively (p = 0.047). However, no difference was present in ratio changes over time. When tested in a multiple regression analysis model including gender, active smoking status, CPB time and cross-clamp time, adjusting for age or not in the model, none of these predictors remained significant.

Given the correlation of Ang-2 change (T0-T2) on hospital LOS, we explored the baseline predictors of delta T0-T2 Ang-2. In univariate analysis, female gender (p = 0.014), the absence of angiotensin-converting enzyme (ACE) inhibitor as a pre-op medication at home (p = 0.009) and blood transfusion within 6 h of CPB initiation (p = 0.029) were statistically significant (Table 4); none of the other baseline characteristics were significant in univariate analysis. In the multivariate regression analysis, female gender (p = 0.005), the absence of ACE inhibitor medication (p = 0.001), blood transfusion (p = 0.042) and the cross-clamp time (p = 0.001) remain significant variables in the model.

## Discussion

In this study, high post-CPB values of Ang-2, and the change in Ang-2 between baseline and the first postop measurement (delta T0-T2) were associated with increase LOS – an important criterion of outcome in post-CPB patients. Under normal conditions, higher levels of Ang-1 promote quiescence in the vascular endothelium. In inflamed areas, however, Ang-2 levels rise, principally in the endothelial cells and the smooth muscles, thus activating the vascular endothelium. *In vitro* and *in vivo* studies have demonstrated that higher concentrations of Ang-2 are associated with elevated production of TNF-α, nitric oxide, VEGF and hypoxia [21,22]. Although the mechanism(s) responsible for systemic vascular endothelium activation during CPB remain complex and are likely the result of multifactorial insults, here we show that CPB-induced angiopoietin dysregulation, as measured by the Ang-2/Ang-1 ratio, was associated with bypass and ischemic time (clamp time). The ratio of Ang-2/Ang-1 increased significantly immediately post-CPB, not only because of a rise in Ang-2, but also importantly because of a marked decrease in Ang-1, in keeping with a decrease in quiescence.

Angiopoietin may in fact signal a contribution to vascular leakage similar to sepsis [13]. Strong arguments about the effect on endothelial permeability, and the potential use of angiopoietins as biomarkers, came from *ex vivo* experiments using patients’ plasma [16,23]. Koning *et al*. showed that postoperative plasma induces impaired endothelial barrier function, using Electric Cell-substrate Impedance Sensing. On the other hand, Hilbert *et al*. showed identical hyperpermeability results using transendothelial dextran flux, but with more effect when the plasma Ang2/Ang1 ratio was high [16]. In contrast to the finding of Hilbert *et al*. regarding the significant association of IL-6 and Ang-2, we found rather a weak positive correlation between theses biomarkers; interestingly we found a tendency for inverse correlation with Ang-1.

Regarding the effect of surgery, a previously published cohort study of 25 adult subjects, looking exclusively at Ang-2, found a similar correlation as us with the duration of CPB, aortic cross-clamp time and post-CPB lactate levels [18]. Another cardiac surgery cohort of 84 adults, including 53 CPB, reported the association between postoperative Ang-2 and the duration of CPB [17]. Our results not only confirmed ‘ischemic predictors’ suggested by others, but also showed a differential pattern of Ang-2 and Ang-1 changes around CPB.

Although useful for clinical decision-making, biomarkers that predict the potential for a complicated postoperative ICU or hospital course have not been identified to date. Disequilibrium in the expression and production of angiogenic factors have been associated with the severity of inflammation and based on the data presented may inform regarding postop hospital LOS. LOS in ICU was already reported to be associated with Ang-2 postop levels [18,24]. In his series of 48 children, Giuliano *et al*. demonstrated that the level of plasma Ang-2 rises shortly after CPB for cardiac surgery and correlates with positive fluid balance and LOS in the ICU [16,24]. Moreover, Jongman *et al*. reported the association of Ang levels dysbalance in patients that develop acute kidney injury after cardiac surgery [25]. As the reflect of increased permeability, increased Ang-2 was shown in other studies to be associated with respiratory failure and prolonged mechanical ventilation [17,18].

One of the limitations of some of these studies is that baseline Ang-2 levels were not available or considered for comparison. In contrast, in our study, we considered the changes of Ang-2, which take into account the baseline value for each individual patient. It is especially important, since patients with cardiovascular disease, cerebrovascular disease or hypertension are more likely to have higher circulating Ang-2 levels [26–28]. However, based on our data absolute values and the kinetics of change are individual specific. Similarities in the kinetics of response may in the future discriminate patients into early outcomes risk groups, in other words, ‘normal’ responders with short hospital stay and ‘hyper’ responders with prolonged LOS.

This study adds to the existing literature confirming the association between the CPB and angiopoietins changes (delta from baseline), as well as corroborating the relationship between increase of Ang-2 and hospital LOS [18,24].

We had no off-pump patient in our cohort of patients. Uchida *et al.* interestingly reported in his series that the 31 patients with off-pump coronary artery bypass grafting had no changes in their immediate postoperative Ang-2, compared with baseline [17]. In contrast, two recent works comparing on-pump (nonpulsatile) to off-pump (pulsatile) patients, found no significant differences in Ang1 and Ang2 levels, despite a more severe systemic inflammation on-pump [23,29]. It then confirms the incrimination of CPB in the induction of systemic inflammation, but raises the question about its unique contribution to Angiopoietin release. Our data showing discrepancy between IL-6 and Ang-2 levels could support these observations.

The ability to quantify endothelial dysregulation and vascular leakage in critical illness is strongly needed, particularly in the area of fluid resuscitation management, to limit overload [30,31]. We could not identify a correlation between Ang-1, Ang-2 or Ang-2/Ang-1 ratio with fluid administration due to unreliable and insufficient data capture, unlike others [16]. Intriguingly the delta creatinine was inversely correlated with the Ang-2/-1 ratio but we could not interpret this finding.

Regarding the analysis of baseline characteristics and angiopoietin changes, interesting predictors were found. First, active smoking seemed to hinder Ang-2/-1 ratio changes, when alcohol consumption led to higher postop ratio; given the small comparative numbers, interpretation should be cautious and further confirmation are needed. Second, despite data suggesting that Ang-2 release could be affected by statins, we found no differences in Ang-1, Ang-2 or the ratio, between those with or without statins (data not shown) [32]. The predictors of Ang-2 change (increase) between pre-op and post-op were the female gender, the absence of ACE inhibitors medication and transfusions during CPB. The ‘sensibility’ to vascular instability, as reported by higher Ang-2 changes, could be determined by hormonal differences. The medication of ACE inhibitors might protect or desensitize due to bradykinins or other mechanisms. Finally, the transfusion of allogenic blood is an obvious trigger, which tends to be minimized by recent trials [33].

We acknowledge various limitations of our study most importantly the small number of patients, particularly for certain comparison, the incomplete availability of samples and the lack of documentation about fluid accumulation. The daily weight, which could not be reliably measured, and albumin level could have strengthened the association of angiopoietins with presumed vascular leakage.

However, we have found a significant correlation with hospital LOS, which provided clinical significance to these markers. Our conclusions are limited given the measures of Ang-1 and Ang-2 function as surrogate markers for the degree of vascular leakage; quantifying and qualifying vascular leakage in the clinical context remains a challenge to overcome.

## Conclusion

Collectively, our results show that measuring angiopoietins provides clinicians with a signal of endothelium imbalance assessment after CPB. It could be useful in detecting the phenomenon of vascular leakage at early stage and guide interventions. Further studies are warranted to validate these findings and clarify its use as a point of care, particularly in the area of fluid resuscitation management.

## Future perspective

The challenge of fluids management around cardiac surgery and the impact on hospital course remains poorly understood. The ability to measure vascular leakage in order to guide clinicians in their postoperative management is promising with angiopoietins (Ang). Within the next 5 years further studies using Ang as biomarkers, that is, the next morning after surgery, are warranted. Ang could be used as a point of care to monitor interventions (i.e., fluid limitation, diuretics or vascular constrictor) or define a category of patients where the approach should be more aggressive. Alternatively it could be measured routinely early after surgery to inform on the vascular status, in order to help clinicians initiating interventions. Among already used monitoring of organ function (i.e., creatinine, cardiac echo, coagulation, PaO_2_, etc.), Ang could represent the future routine marker of endothelium function in various clinical situations, including surgery, sepsis, chronic dialysis and others.

**Table 1.** **Baseline and demographic characteristics (n =41).**

**Characteristic**	**Results**
Male	32 (78)
Age (years)	65 ± 11
BMI, kg/m^2^	29 ± 9
APACHE II	22 ± 5
Co-morbidities:	
– Diabetes	10 (24)
– Hypertension	30 (73)
– Dyslipidemia	22 (54)
– Obesity	8 (20)
– COPD	3 (7)
– Chronic renal disease	8 (20)
– Active smoking	5 (12)
– Chronic alcohol use	7 (17)
Medications:	
– ASA	41 (100)
– Clopidogrel	4 (10)
– Statin	22 (54)
– ACE inhibitor	13 (32)

Values are displayed as mean ± SD or number (%).

ACE: Angiotensin-converting enzyme; COPD: Chronic obstructive pulmonary disease.

**Table 2.** **Type of surgery, hospital course and outcomes.**

Procedures:	
– CABG	20 (49)
– Valve surgery	20 (49)
– Aorta surgery	5 (12)
– Combined	5 (12)
CPB duration, minutes	145 ± 54
Cross-clamp duration, minutes	118 ± 43
– Lowest temperature, °C	31.5 ± 3.3
– Blood transfusion within 0–6 h of CPB initiation	10 (24)
Postoperative course	
– Vasopressors at ICU admission	10 (24)
– Delta creatinine (μg/ml)^†^	6 ± 17
– Renal replacement therapy	0 (0)
– Duration of ventilation, days	1 (1–11)
ICU length of stay, days	2 (1–17)
Hospital length of stay, days	8 (5–24)
Discharged home	35 (85)
Death	0 (0)

Values are displayed as mean ± SD or number (%).

^†^Immediate postoperative minus baseline.

CPB: Cardiopulmonary bypass.

**Table 3.** **Perioperative mediator's changes.**

**Biomarkers**	**Pre-op (T0)**	**ICU admission (T1)**	**Next day (T2)**
Ang-2 (pg/ml)	487 (206, 956)	1240 (861, 2073)*	1856 (1004, 2737)*
Ang-1 (pg/ml)	6309 (2912, 12,646)	6935 (3192, 9263)	4158 (2163, 7226)*
Ang2/1 ratio	0.07 (0.03, 0.25)	0.20 (0.10, 0.43)*	0.43 (0.16, 0.97)*
IL-1β (ng/ml)	4.5 (3.5, 6.1)	5.8 (5.0, 8.2)*	–
IL-6 (ng/ml)	18 (13.5, 24.5)	153.3 (113.9, 239.9)*	–
S-ICAM (ng/ml)	6582 (4644, 10,403)	8390 (6187, 13,713)	–
GMCSF (ng/ml)	42.1 (31.1, 79.6)	42.1 (31.1, 63.7)	–
MCP-1 (ng/ml)	20.4 (15.8, 37.1)	86.7 (45.3, 228.5)*	–
IL-10 (ng/ml)	19.8 (16.7, 26.6)	35.7 (27.3, 56.0)*	–
IL-1RA (ng/ml)	74.5 (51.9, 110.5)	3499 (2243, 5891)*	–
IL-2 (ng/ml)	9.8 (7.0, 17.9)	9.8 (8.0, 12.8)	–
IL-12 (ng/ml)	11.5 (10.2, 15.0)	18.2 (14.7, 25.1)*	–
IL17 (ng/ml)	6.0 (4.3, 10.7)	6.0 (5.0, 8.4)	–

Values are displayed as mean ± SD or N (%) or median (min-max); *immediate postoperative minus baseline.

Median (IQR); *p < 0.05 compared with pre-op (Wilcoxon rank).

**Table 4.** **Significant categorical predictors of delta Ang-2 in univariate analysis.**

**Predictors**	**Yes**	**No**	**p-value**
**Delta angiopoietin 2 T0-T2 (pg/ml), median (IQR)**			
Female	1974 (1642, 3537)	1275 (611, 1684)	0.014
Pre-op ACE inhibitor	875 (-4.6, 1477)	1619 (782, 2092)	0.009
Blood transfusion	1988 (1313, 2333)	1147 (676, 1619)	0.029

Baseline categorical variables present in Table 1 were all tested. Only the significant are displayed.

ACE: Angiotensin-converting enzyme.

**Executive summary**

**Studied biomarkers**

Angiopoietin-1 (Ang-1) and -2 (Ang-2) are biomarkers of endothelial dysfunction.
Clinical context and population.
The phenomenon of vascular inflammation and leakage that occurs in the context of cardiac surgery, with cardiopulmonary bypass (CPB), provides a unique experimental model in humans.
Prospective observational study including adult patients undergoing elective CPB surgery.

**Angiopoietin kinetic**

Circulating Ang-2 levels increased early postoperatively, while in parallel, Ang-1 levels continued to decrease.
The ratio of Ang-2/Ang-1 also rose significantly over time.
Association with other mediators.
A weak positive association of Ang-2 with postoperative IL-6 and an inversed association of Ang-1 with IL-6 were found.

**Clinical associations**

We confirmed that the association of angiopoietin changes and CBP/cross-clamp time.
A significant correlation was found between hospital length of stay and Ang-2 increase at 24 h.
Baseline predictors of Ang-2 increase were female gender, the absence of ACE inhibitor medication, blood transfusion, besides the cross clamp time.

**Conclusion**

Measuring angiopoietins provides clinicians with a signal of endothelium dysfunction after CPB, which could be useful in detecting the phenomenon at early stage and guide interventions.
